# Tuberculosis of the Genito-Urinary Organs

**Published:** 1898-12

**Authors:** 


**Tuberculosis of the Genito-Urinary Organs.** By N. Senn, M.D. Pp. vi., 17—317. London: The Rebman Publishing Co. (Ltd.). Philadelphia: W. B. Saunders. 1898.

We agree with Dr. Senn when he says, in his preface to this work, that the time has come when a special treatise on this subject will fill a gap in medical literature. So much has lately been written about genito-urinary tuberculosis, that such a book as this, bringing to a focus the information from all sources, and adding much from the author's own experience, is of no little value. It is not only to the surgeon that this work appeals: to the physician tuberculosis of the kidney presents difficulties in diagnosis; and to the gynæcologist we can recommend the study of the portion of the book which relates to tuberculosis of the female organs of generation, and this constitutes a larger proportion of the work than might be supposed. Tubercular lesions of the Fallopian tubes are constantly overlooked, for, as the author states, it often requires a very careful histological and bacteriological examination to detect the true nature of the disease.

One thing which strikes the reader of this book is the uncertainty which exists as to the frequency with which tubercle starts in particular organs, especially whether it more frequently begins in the kidney or bladder. The author is not able to give any satisfactory explanation of the well-recognised fact that when tubercular disease affects the testicle, it begins in the epididymis rather than the testis proper. We must say we are disappointed at the way so important a subject as the differential diagnosis of tuberculous testis from other forms of induration of the epididymis is considered. No mention is made of the induration left by the ordinary epididymitis which may simulate tuberculous disease, or of the rare form of syphilitic epididymitis to which several writers have of late years called attention. Then again the statistics on pages 75 and 76 of the results of castrations for tuberculous testis are not satisfactory. They fail to give us just the one fact we want most to know—how long the patient remains free from any further tuberculous disease in the genito-urinary organs after castration.

The reference to several operations for excision of tuberculous vesiculæ seminales, and the report of Fenger's case, are very interesting; and the successful results obtained in two cases of primary tuberculous disease of the prostate by scraping out the broken-down tubercular material is encouraging.

The drawings of the histology of the tuberculous lesions in various organs are very good, but perhaps hardly necessary, as they all show the familiar tubercles; but the coloured plate of tuberculous disease of the kidneys is one of the best of a diseased organ we have seen.

The book is well worth careful study; but the reader must expect to find that much difference of opinion exists about genito-urinary tuberculosis, and to be left in doubt as to the best treatment to adopt. The profession is, however, much indebted to Dr. Senn for his labour in the production of a work which so faithfully reflects these different views.

